# Serum miRNA Signature in Rheumatoid Arthritis and “At-Risk Individuals”

**DOI:** 10.3389/fimmu.2021.633201

**Published:** 2021-03-03

**Authors:** Clare C. Cunningham, Sarah Wade, Achilleas Floudas, Carl Orr, Trudy McGarry, Siobhan Wade, Sian Cregan, Ursula Fearon, Douglas J. Veale

**Affiliations:** ^1^ Molecular Rheumatology, School of Medicine, Trinity College Dublin, Dublin, Ireland; ^2^ EUropean League Against Rheumatism (EULAR) Centre of Excellence, Centre for Arthritis & Rheumatic Diseases, University College Dublin, Dublin, Ireland

**Keywords:** rheumatoid arthritis, microRNA, inflammation, arthralgia, therapy, at-risk individuals

## Abstract

**Background:**

MicroRNAs (miRNAs) are small non-coding RNAs which have been implicated as potential biomarkers or therapeutic targets in autoimmune diseases. This study examines circulatory miRNAs in RA patients and further investigates if a serum miRNA signature precedes clinical manifestations of disease in arthralgia or “at-risk individuals”.

**Methods:**

Serum was collected from HC subjects (N = 20), RA patients (N = 50), and arthralgia subjects (N = 10), in addition to a subgroup of the RA patients post-methotrexate (MTX) (N = 18). The FirePlex miRNA Immunology-V2 panel was selected for multiplex analysis of 68 miRNAs in each sample. DNA intelligent analysis (DIANA)-mirPath and Ingenuity Pathway Analysis (IPA) software were used to predict pathways targeted by the dysregulated miRNAs.

**Results:**

8 miRNA (miR-126-3p, let-7d-5p, miR-431-3p, miR-221-3p, miR-24-3p, miR-130a-3p, miR-339-5p, let-7i-5p) were significantly elevated in RA serum compared to HC (all p < 0.01) and 1 miRNA (miR-17-5p) was significantly lower in RA (p < 0.01). High specificity and sensitivity were determined by receiver operating characteristic (ROC) curve analysis. Both miR-339-5p and let-7i-5p were significantly reduced post-MTX (both p < 0.01). MiR-126-3p, let-7d-5p, miR-431-3p, miR-221-3p, miR-24-3p, miR-130a-3p were also significantly elevated in subjects “at risk” of developing RA (all p < 0.05) compared to HC. IPA analysis of this miRNA signature identified downstream targets including key transcription factors NF-κB, STAT-1, STAT-3, cytokines IL-1β, TNF-α, and matrix-metalloproteases all importantly associated with RA pathogenesis.

**Conclusion:**

This study identified six miRNAs that are altered in both RA and “at-risk individuals,” which potentially regulate key downstream pathways involved in regulating inflammation. These may have potential as predictive signature for disease onset and early progression.

## Introduction

MicroRNAs (miRNAs) are small non-coding RNAs which play an important role in numerous biological processes such as cell differentiation and homeostasis, through the regulation of gene expression (1). Since their discovery, they have been implicated in cancer, viral, neurodegenerative and autoimmune diseases (2). Binding to complementary sequences on messenger RNA (mRNA), miRNAs generally function to suppress the translation of target proteins, however, they have also been shown to control the rate of transcription. Furthermore, under certain conditions and in specific cell types, they can, in fact, induce gene expression (3). While the genetic predisposition to rheumatoid arthritis (RA) has been recognized, new discoveries continue to reveal specific genes and mutations linked to RA pathogenesis and, in the last 10 years, miRNAs have also been implicated (4). A number of studies have reported that dysregulated miRNA expression influences immune regulation, enhances pro-inflammatory signaling pathways, and leads to the overproduction of pro-inflammatory cytokines in RA (5–9).

In addition to their localization within the cell, miRNAs are also present in extracellular fluids such as serum, plasma, and synovial fluid, and can be transported to target cells and tissues through the circulation, thus acting as signaling molecules for intercellular communication (10–12). These are more stable than cellular miRNAs and many have been reported as potential biomarkers for disease (13, 14). Early diagnosis is crucial in halting the development of RA in order to prevent disability and maintain quality of life, and systemic inflammation is often detected in individuals prior to the clinical presentation of arthritis. The presence of markers such as anti-citrullinated protein antibodies (ACPA) and Rheumatoid Factor (RF) have proven to be useful early diagnostic markers for “at-risk individuals” (15) and recent studies have suggested that the presence or absence of circulating miRNAs may also pre-empt the development of arthritis or reflect different disease stages (16, 17). Furthermore, the response to treatment varies greatly between patients, and the expression of specific miRNAs could explain this and, in turn, help clinicians select a suitable course of treatment (18–20).

Therefore, this study evaluated the expression profile of circulating miRNAs, specifically focusing on a defined immunology miRNA panel, in patients with RA and in “at-risk individuals,” and assessed the change in expression levels following methotrexate (MTX) treatment. This study identified eight miRNAs that are elevated and one that is decreased in the circulation of RA patients compared to healthy controls (HC) which may serve as prognostic biomarkers for disease development in “at-risk individuals” or may be predictive of response to MTX treatment.

## Methods

### Patient Recruitment

Fifty patients with RA were recruited from the outpatient clinic at the Department of Rheumatology, St. Vincent’s University Hospital. Patients fulfilled the American College of Rheumatology (ACR)/European League Against Rheumatism (EULAR) classification criteria for RA (21, 22). One patient’s serum miRNA were outside the detection limit of assay so were excluded. A cohort of 18 patients were prescribed MTX and were assessed at baseline and at a follow-up appointment, 3 months post-treatment, for response based on ACR/EULAR response criteria (21, 22). Patient demographics are included in **Supplementary Tables 1** and **2**. A second blood sample for serum miRNA analysis was also taken at this time point. Serum was also obtained from a cohort of arthralgia or “at-risk individuals” (N = 10), defined as subjects with symptoms of aches and pains and with positive circulating RF and ACPA, but without clinical signs of synovitis or raised CRP (<5 mg/L) (23), with a median age of 49 (27–68 years) and F:M 8:2, patient demographics **Supplementary Table 1**. HC individuals (N = 20) were also assessed. All research was performed in accordance with the Declaration of Helsinki, and ethical approval for this study was granted by the St. Vincent’s University Hospital Medical Research and Ethics Committee (ref-RS-18-055). All patients gave fully informed written consent prior to inclusion.

### Serum miRNA Expression Analysis 

Patient blood was collected in anticoagulant-free tubes and processed within 1 h of collection as follows: samples were centrifuged at 2,000 g for 10 min at room temperature to isolate the serum which was transferred to RNase- and DNase-free tubes. The FirePlex Circulating miRNA Immunology Fixed Panel **(** **Supplementary Table 3** **)** (Abcam, Cambridge, UK) was selected for multiplex miRNA analysis of 68 miRNAs in serum as previously described (14). This panel was utilized to focus on miRNA associated with immune cell dysfunction in autoimmune diseases, and included miRNA that have been previously identified to be associated with RA, either by expression in serum/tissue/cells or associated with immune cell/synovial fibroblast dysfunction (14, 17–20, 24). Specifically we utilized this technique to assess the utility of the FirePlex as a high-throughput platform which can reliably quantify miRNA directly from crude biofluids with minimal sample preparation, thus reducing the RNA isolation-induced variability.

Briefly, serum miRNA were hybridized to miRNA probes by incubation of 20 μl of patient serum with 35 μl of supplied FirePlex hydrogel particles and 25 μl of hybridization buffer with gentle agitation for 60 min at 37°C. Samples were rinsed twice with supplied rinse buffer A prior to addition of 75 μl of labeling mix containing supplied labeling diluent, labeling buffer, and labeling enzyme followed by incubation with gentle agitation for 60 min at room temperature. Samples were rinsed twice with supplied rinse buffer B and once with rinse buffer A prior to addition of 110 μl of RNase-free water and incubation with gentle agitation for 30 min at 55°C. Ligated miRNAs were eluted and amplified by PCR using the supplied PCR buffer, dNTP mix, PCR enzyme, and biotin-labeled primer mix, using a single step universal primer RT-amplification consisting of 27 cycles of PCR amplification followed by six cycles of asymmetric amplification. Amplified products were then re-hybridized with FirePlex particles in 75 μl of reporting buffer and incubated with gentle agitation for 15 min at room temperature. Following addition of 175 μl of run buffer, particle-bound miRNA were detected on an EMD Millipore Guava 8HT flow cytometer. Internal positive, negative, and blank controls were included to validate assay performance and correct for background signals. Analysis was performed in R (v4.0) using package Seurat (v3.2.3), function scale and package gplots (v3.1.1), function heatmap2. Distance matrix was generated by Euclidian distance measure and clustering performed with complete agglomeration. PCA analysis was performed in R (v4.0) function prcomp with scaling. Raw data from the FirePlex platform is available on request.

### Bioinformatic Analysis

The DNA intelligent analysis (DIANA)-mirPath online software suite (25) was used to predict the Kyoto Encyclopedia of Genes and Genomes (KEGG) pathways potentially altered by the dysregulation of the miRNAs identified in this study. Pathway exploration of the dysregulated miRNAs was performed and visualized using QIAGEN Ingenuity Pathway Analysis (IPA) software (https://www.qiagenbioinformatics.com/products/ingenuity-pathway-analysis) (26).

### Statistical Analysis 

Intergroup differences were deemed significant based on their Bonferroni-corrected adjusted p-value, calculated using the FirePlex Analysis Workbench software. Nine miRNAs were shown to be significantly different between RA and HC. Subsequent statistical analysis was performed using GraphPad Prism 8 software. The nonparametric, Mann-Whitney *U* test was used to determine significant differences between RA and HC or between arthralgia and HC. Wilcoxon matched-pairs signed rank test was used to determine significant differences between RA patients pre- and post-MTX treatment. P ≤ 0.05 was deemed statistically significant. Receiver operating characteristic (ROC) curves displaying area under the curve (AUC) with 95% confidence interval (CI) were constructed using GraphPad Prism 8 software to demonstrate the diagnostic ability of a selected miRNA to predict disease or response to MTX.

## Results

### Rheumatoid Arthritis Serum Displays a Distinct miRNA Profile Compared to Healthy Serum

**Figure 1:**
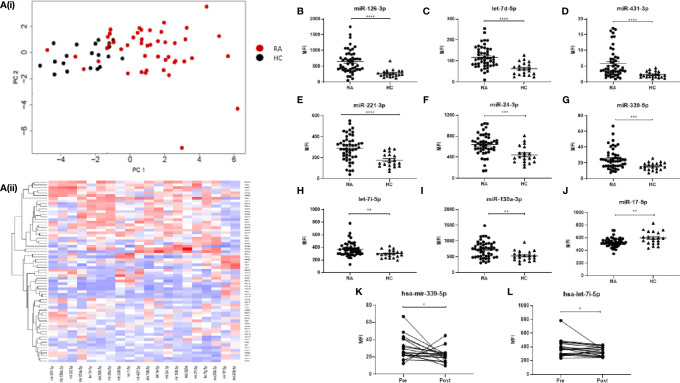
**(A)** Hierarchical clustering analysis of miRNA expression for the top 20 differentially expressed miRNAs between RA and HC. (i) PCA plots and (ii) Heatmap depicting serum miRNA expression in 49 RA patients and 20 HC individuals. Analysis was performed in R (v4.0) using package Seurat (v3.2.3), function scale and package gplots (v3.1.1), function heatmap2. Distance matrix was generated by Euclidian distance measure and clustering performed with complete agglomeration. PCA analysis was performed in R (v4.0) function prcomp with scaling. **(B–J)** Expression of 9 miRNAs of interest between RA patients and HC individuals. miRNA expression was determined by Multiplex Circulating miRNA Assay. Graphs show mean fluorescence intensity (MFI) for RA patients (N = 49). HC individuals (N = 20), and **(K, L)** for RA patients pre- and 3 months post-MTX treatment (N = 18), represented as mean ± SEM. ****p ≤ 0.0001, ***p≤ 0.001, **p ≤ 0.01, *p ≤ 0.05 as determined by Mann-Whitney *U* test.

In order to compare the miRNA signature of RA and healthy sera, a specific panel of 68 miRNAs, known to be associated with immune dysfunction (14), was selected for multiplex miRNA analysis of serum from HC donors (N = 20) and RA patients that were naïve to treatment (N = 50). The FirePlex Analysis Workbench software was used to analyze and determine significant differences in miRNA expression, between RA and HC, and generate heatmaps. **Figures 1Ai–ii** represent the top 20 differentially expressed (all p < 0.01) miRNAs between RA and HC. Using PCA analysis and hierarchical clustering, we demonstrated that specific RA patients clustered separately HC, with some overlap, as show in the PCA plots.

Further analysis identified eight miRNAs of interest (miR-126-3p, let-7d-5p, miR-431-3p, miR-221-3p, miR-24-3p, miR-339-5p, miR-130a-3p, let-7i-5p) which were significantly elevated (**Figures 1B–I**) (all p ≤ 0.01) in RA compared to HC, and one miRNA (miR-17-5p) which was significantly lower in RA (p = 0.0061; **Figure 1J**). Of the 50 naïve RA patients, a cohort of 18 was prescribed MTX, a front-line disease-modifying anti-rheumatic drug (DMARD) and a second blood sample was taken for serum miRNA analysis at a follow-up appointment 3 months post-treatment. Clinical parameters of patients pre- and post-MTX are shown in **Supplementary Table 2**. From the nine miRNAs of interest, only miR-339-5p and let-7i-5p were significantly altered (p = 0.0052, p = 0.0335 respectively) by MTX treatment (**Figures 1K, L**).

**Figure 2:**
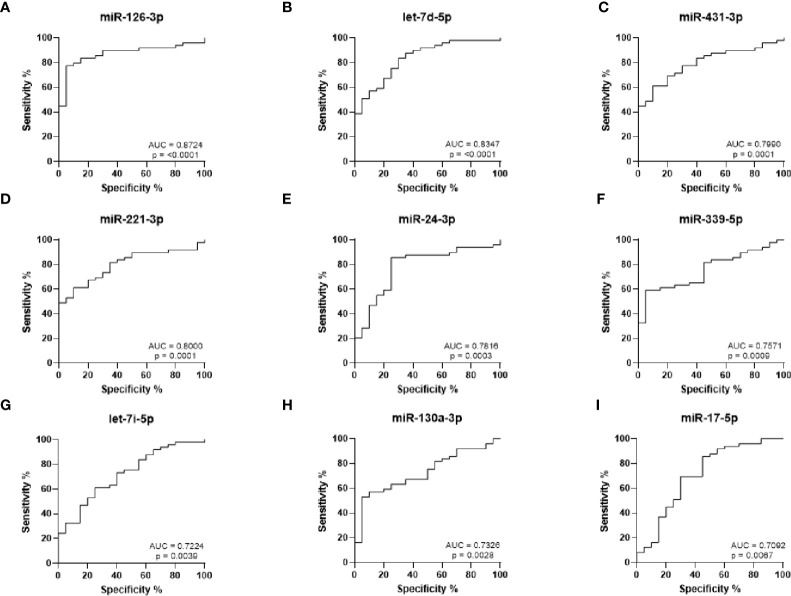
ROC curve analysis for RA diagnosis based on miRNA expression levels. **(A–I)** ROC curves showing the prediction accuracy for each miRNA to differentiate between RA and HC. AUC calculated with 95% confidence intervals.

ROC curves were generated to analyze the sensitivity and specificity of each miRNA to discriminate between RA and HC (**Figures 2A–I**). The strongest statistical separation was observed for miR-126-3p with an AUC of 0.8724 (p < 0.0001), while both miR-221-3p and let-7d-5p also had an AUC ≥ 0.8 (p ≤ 0.0001). This suggests that these miRNAs may be useful biomarkers for RA.

### Serum miRNA Expression Increases Prior to Rheumatoid Arthritis Onset

**Figure 3:**
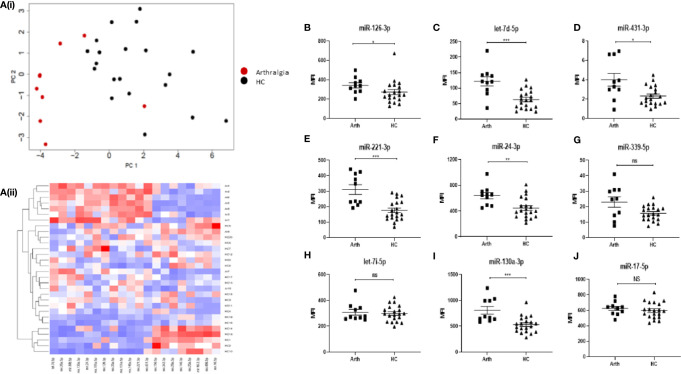
**(A)** Hierarchical clustering analysis of miRNA expression for the top 20 differentially expressed miRNAs between arthralgia and HC. (i) PCA plots and (ii) Heatmap depicting serum miRNA expression in 10 Arthralgia and 20 HC individuals. Analysis was performed in R (v4.0) using package Seurat (v3.2.3), function scale and package gplots (v3.1.1), function heatmap2. Distance matrix was generated by Euclidian distance measure and clustering performed with complete agglomeration. PCA analysis was performed in R (v4.0) function prcomp with scaling. **(B–J)** Expression of nine miRNAs of interest between arthralgia patients and HC individuals. miRNA expression was determined by Multiplex Circulating miRNA Assay. Graphs show MFI for arthralgia patients (N = 10) and HC individuals (N = 20), represented as mean ± SEM. ***p ≤ 0.001, **p ≤ 0.01, *p ≤ 0.01 and NS, not significant as determined by Mann-Whitney *U* test.

In order to determine whether miRNA expression can be used as a biomarker to predict disease development, blood samples were also taken from a cohort of 10 “at-risk individuals” who present with joint pain (arthralgia), but do not show clinical signs of synovitis or raised CRP. The heatmap in **Figures 3Ai–ii** show the top 20 differentially expressed (all p < 0.01) miRNAs between arthralgia and HC. Using PCA analysis and hierarchical clustering, we demonstrated that arthralgia patients clustered separately from HC, with some overlap, as show in the PCA plots.

Interestingly, six of the miRNAs (miR-126-3p, let-7d-5p, miR-431-3p, miR-221-3p, miR-24-3p, and miR-130a-3p) that are elevated in RA compared to HC, as reported in **Figure 1**, are also significantly elevated in arthralgia (**Figures 3B–J**), with let-7d-5p miR-221-3p and miR-130-3p being the most significantly different.

**Figure 4:**
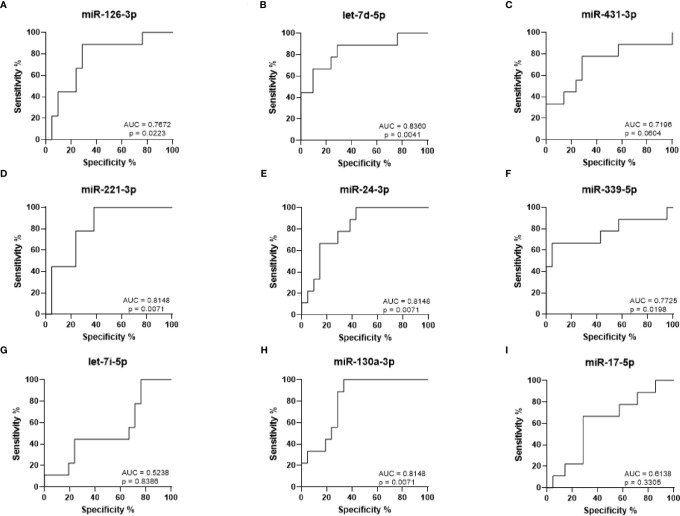
ROC curve analysis for RA diagnosis based on arthralgia miRNA expression levels. **(A–I)** ROC curves showing the prediction accuracy for RA conversion based on miRNA expression levels. AUC calculated with 95% confidence intervals.

ROC curves were generated to analyze the sensitivity and specificity with which the levels of each miRNA can be used to discriminate between arthralgia and HC (**Figures 4A–I**). The strongest statistical separation was observed for let-7d-5p with an AUC of 0.8360 (p = 0.0041), followed by miR-221-3p, miR-130a-3p, and miR-24-3p, all with an AUC of 0.8148 (p = 0.0071). For Arth v HC, mir-130a-3p in combination with either let-7d-5p, miR-221-3p, miR-24-3p, miR-431-3p, and miR-126-3p gives a higher AUC than any of them alone. Triple combination of mir-130a-3p and let-7d-5p with either miR-221-3p, miR-24-3p, or miR-126-3p gives an AUC > 0.9000 (**Supplementary Figure 1**). This suggests that elevated expression of these miRNAs may be predictive of disease onset.

Furthermore, we analyzed previously identified miRNA known to be associated with RA including miR-146, miR-125a/b, miR-16, miR-223, miR-155. MiR-146 and miR223 were significantly different in RA compared to HC, however these weren’t as significant as the nine miRNA identified (**Supplementary Figure 2**). miR-146 was also upregulated in Arthralgia (**Supplementary Figure 2**). MiR-125a-5p and miR-16-5p were significantly increased in RA *vs* Arthralgia, but not HC, while no change was observed for miR-155 (**Supplementary Figure 2**).

### Functional Pathway Analysis of Dysregulated miRNAs

**Figure 5:**
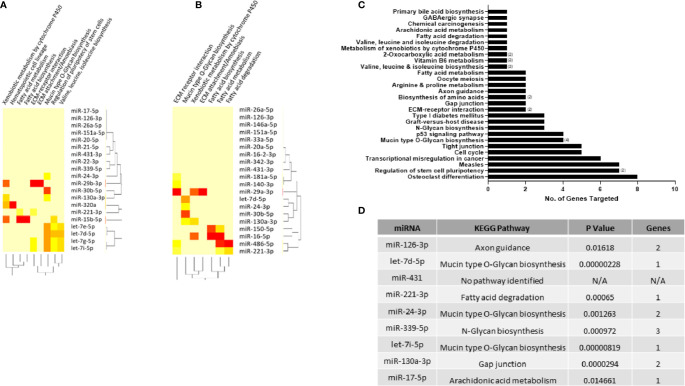
KEGG pathways enriched in miRNA target genes. DIANA-miRPath tool was used to identify KEGG pathways enriched in genes significantly targeted by the top 20 differentially expressed miRNAs in **(A)** RA and **(B)** arthralgia compared to HC. Heatmap based on significance (log p value) of the interaction between each miRNA (rows) and the target pathway (columns) as determined by Fisher’s Exact test. DIANA-miRPath tool was used to identify KEGG pathways enriched in genes significantly targeted by the nine miRNAs of interest. **(C)** Bar graph represents the number of genes in each pathway targeted by one or more (number in brackets) miRNA. **(D)** Table shows top pathway targeted by each of the nine miRNAs of interest.

As a particular miRNA may act on several targets, the DIANA miRPath tool (25) was used to identify KEGG pathways that are enriched in genes containing predicted target sites for the top 20 miRNAs found to be dysregulated in RA and arthralgia serum compared to HC. Fisher’s Exact Test with a p value threshold of 0.05 was used to determine which pathways were significantly targeted and generate heatmaps for RA (**Figure 5A**) and arthralgia (**Figure 5B**). Fatty acid biosynthesis and extracellular matrix (ECM) receptor interaction were the topmost significantly targeted pathways by both RA and arthralgia serum miRNAs. Additionally, fatty acid metabolism, mucin-type O-glycan biosynthesis, ECM attachment/amoebiasis, and xenobiotic metabolism by cytochrome P450 were pathways common to both. Arthralgia serum miRNAs also significantly target the fatty acid degradation pathway, while RA serum miRNAs significantly targeted pathways associated with valine, leucine, and isoleucine metabolism. **Figure 5C** shows the pathways, and the number of genes within each, that are targeted by the nine miRNAs of interest to this study. Interestingly miR-24-3p alone potentially targets eight genes in the osteoclast differentiation pathway. **Figure 5D** shows the most significant pathway targeted by each miRNA with mucin type O-glycan biosynthesis being the most significantly targeted by three of the nine miRNAs. This shows that the pathways targeted by the dysregulated miRNAs may play direct and indirect roles in the pathogenesis of RA.

**Figure 6:**
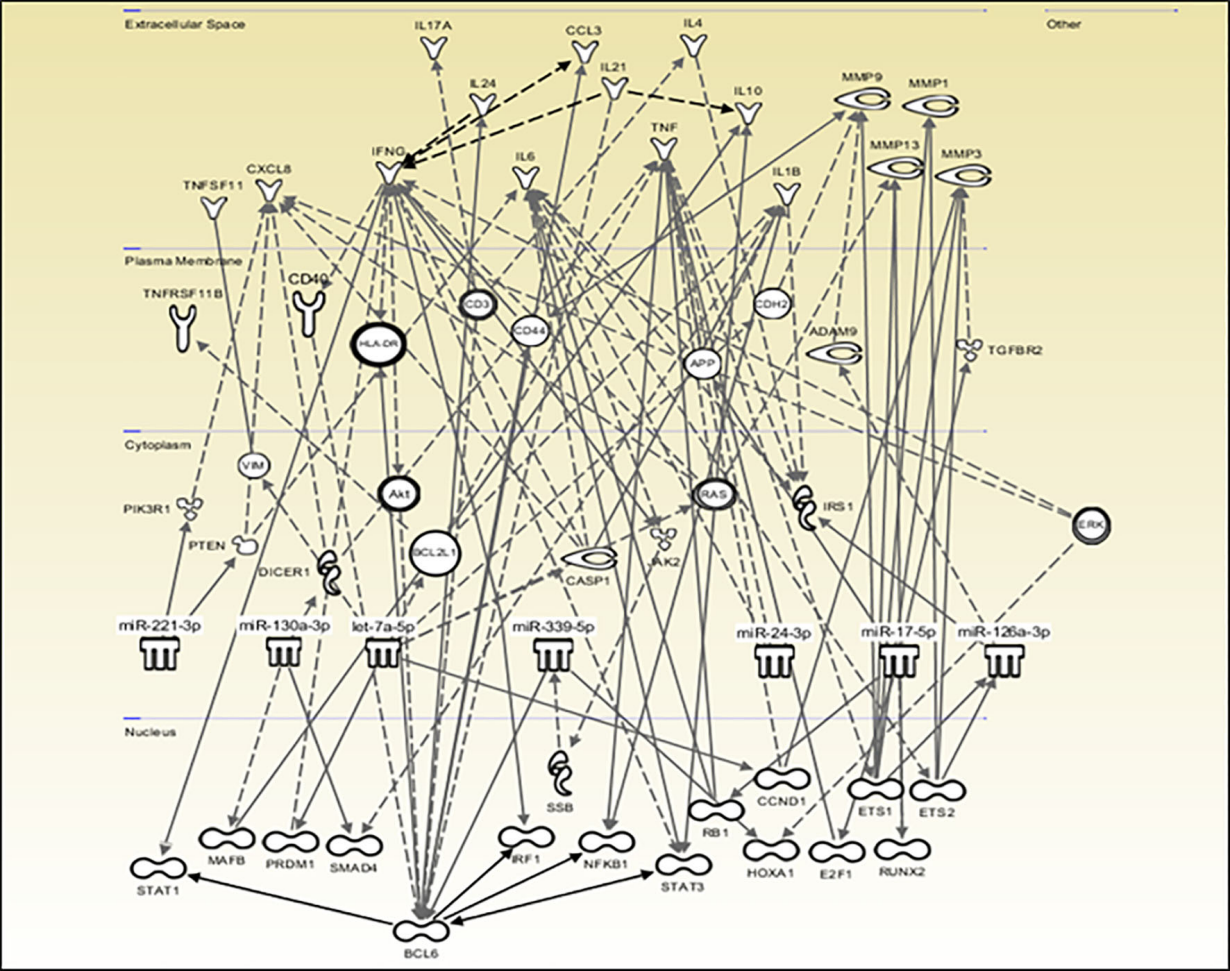
Predicted potential miRNA interactions and downstream effects. QIAGEN IPA software was used to explore and visually represent the potential role that the nine miRNAs of interest may play in RA based on direct and indirect interactions which may impact the production of downstream effector molecules. miR-431-5p did not significantly target any pathway and was omitted from the diagram. let-7d-5p and let-7i-5p are grouped under the let-7a-5p symbol.

The QIAGEN IPA software was used to create a network of potential direct and indirect interactions between the miRNAs and downstream molecules such as transcription factors, receptors, enzymes, and cytokines, allowing the visualization of the data in the context of biological systems **(** **Figure 6** **)**. Consistent with the DIANA results, miR-431-5p did not significantly target any pathway and is therefore absent from the network while let-7d-5p and let-7i-5p are grouped under the let-7a-5p symbol. This network shows the involvement of transcription factors such as BCL6, IRF1, NF-κB, STAT-1, and STAT-3, and the downstream production of cytokines and degradative enzymes such as IL-1β, TNF-α, IL-17, and MMP-1, -3 and -9, all of which are associated with RA.

## Discussion

The identification of reliable serum biomarkers for the early diagnosis of RA is an area of focused investigation, with studies demonstrating that the earlier that patients are treated, the better the response and outcome (27). However, to date, other than ACPA positivity, no validated biomarkers are utilized in the clinical setting. In the present study, we identified a specific group of circulatory miRNA as potential biomarkers of RA. Specifically, this study focused on a panel of miRNA associated with immunological function and demonstrated a significant increase in expression of eight miRNAs in RA compared to HC, two of which were significantly reduced at 3 months post-MTX therapy. Furthermore, analysis of arthralgia subjects demonstrated a significant increase in six of these miRNA, suggesting their potential to support early diagnosis of RA disease development.

RA disability stems from structural damage of cartilage and bone, due to erosions in synovial joints, if not treated early and aggressively. Treatment guidelines are based on clinical factors including RF and, more recently, ACPA, which may predict outcome. Indeed, these antibodies may be present before the onset of clinical arthritis; in some cases, several years before, suggesting that autoimmunity precedes inflammation (28). These “at-risk of arthritis” or “pre-RA arthralgia” subjects have been the focus of many studies in recent years and may provide important clues in understanding the evolution of RA and identifying biomarkers to predict disease onset. One potential corollary is the promise of developing a cure for RA where the initial break in self-tolerance is identified early and targeted therapeutically. Although ACPA+ arthralgia is associated with subsequent development of RA in some subjects, the conversion rate varies greatly between 30 and 70%. A few studies have observed altered immune cell function in these “at-risk” populations, suggesting pre-existing autoimmune dysregulation, however, blood or tissue biomarkers have not, as yet, been identified (29, 30). Serum miRNAs are stable and may be increased in the circulation due to their shedding from a site of disease or, alternatively, are used as a mechanism of communication between distal cells and tissues via the circulation and have thus been reported as potential biomarkers for numerous diseases (10–14). Based on these studies, we hypothesized that circulating miRNAs may be utilized as potential biomarkers of both RA and ACPA+ “pre-RA”.

MiRNAs have been implicated in many diseases including cancer, viral infections such as Hepatitis C virus and neurodegenerative diseases such as Alzheimer’s disease (5). However, it is their role in immune-related diseases that is particularly relevant to the field of RA research (31) ; to date, the most prominent miRNAs associated with RA are miR-146 and miR-155, both of which are implicated in the regulation of the NF-κB pathway, a pivotal mediator of inflammation, in addition, both miRNAs are shown to be elevated in RA synovial tissue and circulating immune cells compared to HC and osteoarthritis (OA) (31–36). In this study, using hierarchical clustering, we identified differentially expressed serum-derived miRNA that distinguished RA patients from HC. Further analysis identified 8 miRNAs, associated with immunological function, which were significantly elevated in RA compared to HC (miR-126-3p, let-7d-5p, miR-431-3p, miR-221-3p, miR-24-3p, miR-339-5p, miR-130a-3p, let-7i-5p), with one miRNA (miR-17-5p) significantly reduced. Two miRNA, miR-339-5p and let-7i-5p, were also significantly reduced following MTX therapy. Given that miRNAs may promote pro-inflammatory processes, this observed reduction may reflect reduced inflammation as a result of MTX treatment. We also examined these miRNA in ACPA+ “at-risk individuals” and demonstrated that six miRNAs (miR-126-3p, let-7d-5p, miR-431-3p, miR-221-3p, miR-24-3p, and miR-130a-3p) of the nine miRNAs identified in the RA cohort, were also significantly elevated in ACPA+ “at-risk individuals,” suggesting that these are present in the circulation prior to disease onset. Furthermore, ROC curves demonstrated that miR-126-3p, let-7d-5p, and miR-221-3p had the highest diagnostic capability for RA and, of these, let-7d-5p and miR-221-3p also showed high statistical separation for “at-risk individuals” and therefore may be useful as predictive biomarkers for the risk of RA development.

Let-7d-5p, miR-431-3p, miR-24-3p, miR221-3p, mi-126-3p and miR339-5p have been implicated in inflammatory diseases and tumor biology but also in (24, 37–41). MiR-24-3p and mi-126-3p is reported to be elevated in the plasma of RA patients compared to OA and systemic lupus erythematosus patients (39), while overexpression of miR-431-5p was recently reported to inhibit cell proliferation and promote apoptosis of RA synovial fibroblasts *in vitro.* However, *in vivo*, miR-431-5p expression was, in fact, downregulated in RA synovial tissue and fibroblasts, thus allowing these processes to proceed and potentially contribute to RA pannus formation (40). MiR-221-3p is elevated in both psoriatic arthritis and RA serum (14, 41), with *in vitro* studies demonstrating that its downregulation suppresses migration, invasion, and pro-inflammatory cytokine production by RA fibroblasts through the inhibition of vascular endothelial growth factor (VEGF) and MMPs (41). MiR-126 is also highly expressed in vascular endothelial cells and is associated with promoting angiogenesis by favoring the proliferation and migration of endothelial progenitor cells and repressing negative regulators of the VEGF pathway (42). Furthermore miR-130a is downregulated in the PBMC of patients with ankylosing spondylitis and *in vitro* studies showed that inhibition of miR-130a increases TNF-α expression, while increased miR-130a inhibits apoptosis and promotes proliferation of T cells (43, 44). Collectively, these miRNA may play a role in regulating angiogenesis, T cell activation, and synovial fibroblast activation, all key mechanisms involved in RA pathogenesis.

Two members of the let-7 family; let-7d-5p and let-7i-5p, were found to be elevated in RA serum compared to HC, with let-7d-5p also significantly elevated in arthralgia serum. Furthermore, a significant reduction in the expression of let-7i-5p following 3 months of MTX treatment was observed, suggesting additional potential for this miRNA as a predictor of response to treatment. These miRNA are members of the let-7 family of miRNA which have been implicated in the development of various cancers due to their role in cell proliferation, a hallmark of tumor development (45–47), however few studies to date have examined their levels or function in RA. Studies have demonstrated elevated levels in ankylosing spondylitis T-cells, with levels correlating with disease activity (48), in addition to increased levels also identified in patients with scleroderma (49).

Numerous studies have demonstrated altered levels of miR-146, miR-125a/b, miR-16, miR-223, and miR-155 in RA, levels of which are associated with RA disease activity and/or regulate pathogenic mechanisms involved in driving inflammation (50–58). Consistent with these studies we also demonstrated a significant increase in miR-146 and miR-223 in RA and arthralgia compared to HC (31, 35, 51, 52), however these weren’t as significant as the nine miRNA identified. In addition both MiR-125a-5p and miR-16-5p were significantly increased in RA *vs* Arthralgia, consistent with previous studies demonstrating higher levels in established RA and PsA (14, 53–56), with studies showing that they also mediate RAFLS function, macrophage plasticity and regulates pro-apoptotic and metabolic pathways in CD14+ monocytes (14, 53–57). Furthermore, miR-125b is also associated with good clinical response in RA patients 3 months post rituximab treatment (58). Interestingly no change was observed for miR-155, which has previously been shown to be elevated in various different cell-types in RA, in addition to influencing B cell function and monocyte apoptotic mechanism (33, 34, 36, 50). These differences may be due to heterogeneity of patient cohorts, in addition miRNA analysis can depend on the site and cell type, with studies also showing an inverse relationship in miRNA levels between systemic and local inflammation (56).

Of the nine miRNAs of interest in this study, miR-17-5p was the only miRNA to be downregulated in RA compared to HC serum. The miR-17-92 cluster has been implicated in driving inflammation in RA (59). Consistent with our findings, Najm *et al.* reported decreased miR-17-5p in erosive RA synovial tissue and demonstrated that miR-17 transfection into arthritic paws in a collagen-induced arthritis model reduced inflammation, immune cell infiltration, and structural damage and identified STAT-3 and JAK-1 as targets of miR-17 in RA synovial fibroblasts (60). Furthermore, Akhtar et al. reported that miR-17-5p is reduced in RA serum and synovial tissue, inversely correlating with RA pathogenesis, and that overexpression of miR-17-5p inhibited TNF-α-induced production of IL-6, IL-8 and MMPs, suggesting that reduction of miR-17-5p expression may restore these pro-inflammatory and degradative processes (61).

Functional pathway analysis of the top 20 dysregulated miRNAs identified fatty acid biosynthesis as the most significantly targeted pathway by both RA and arthralgia serum miRNAs, which may be indicative of the altered bioenergetics observed in RA (62, 63). Additional genes potentially targeted by these miRNAs are associated with osteoclastogenesis and ECM-receptor interaction pathways, including collagen and laminin known to be involved in erosive processes in RA (64, 65). The mucin type O-glycan biosynthesis pathway was the most significantly targeted pathway by three (let-7d-5p, let-7i-5p and miR-24-3p) of the nine miRNAs of interest in this study. As changes in glycosylation of IgG and IgA precede RA onset and correlate with disease severity, increased expression of these miRNAs may be reflective of such pathogenic changes (66, 67). Of particular interest is the network of interactions through which these miRNA may influence the production of downstream effector molecules. IPA identified STAT-1, STAT-3, IRF-1, NF-κB, and BCL-6 amongst the transcription factors directly or indirectly targeted by RA and arthralgia miRNAs. It is well known that these pathways are associated with the production and activity of numerous destructive and inflammatory mediators including the cytokines, IL-1, IL-17, IL-6, IFN-γ, and matrix-degrading enzymes, MMP-1, MMP-3 and MMP-9, all of which play a role in RA and were identified in the IPA network.

This study focused on a specific panel of miRNA associated with immune cell dysfunction and did not perform a full miRNA screen, therefore there are some limitations of the study, in that there may be other miRNA that are also associated with RA and Arthralgia that were not included in the panel. Furthermore, additional cohorts would further validate the miRNA signature presented in this study, however in support of our data, previous studies using different technologies have reported dysregulated expression of some of these miRNA in RA (35–54). While our findings are consistent to these studies, larger multicenter studies are required to validate our signature findings. In this study we specifically utilized FirePlex technology as a high-throughput platform which can reliably quantify miRNA directly from crude biofluids thus reducing the RNA isolation-induced variability. Similar technologies have also emerged that also minimize extraction and sample processing and are highly sensitive (68).

In conclusion, the benefit of a serum miRNA signature as a biomarker is that a minimally invasive and straightforward blood test would be sufficient for diagnosis using the FirePlex assay, a reproducible test which requires only minimal serum in order to simultaneously detect a panel of selected miRNAs, without an RNA purification and amplification step. The serum miRNA signature identified in this study discriminated both RA patients and “at-risk individuals” from healthy subjects and therefore may be useful in the diagnosis of disease at an early time point as well as monitoring response to treatment or risk of recurrence in an efficient, convenient, and cost-effective manner.

## Data Availability Statement

The raw data supporting the conclusions of this article will be made available by the authors, without undue reservation.

## Ethics Statement

The studies involving human participants were reviewed and approved by St. Vincent’s Hospital Ethics Committee. The patients/participants provided their written informed consent to participate in this study.

## Author Contributions

SC, SiW, CO and DJV collected the clinical samples, analyzed data, and prepared the manuscript. CC, SaW, TM, AF and SC performed experiments, analyzed data and prepared the manuscript. UF and DJV conceived the experimental approach, analyzed data, supervised and prepared the manuscript. All authors contributed to the article and approved the submitted version.

## Conflict of Interest

The authors declare that the research was conducted in the absence of any commercial or financial relationships that could be construed as a potential conflict of interest.

## Supplementary Material

Supplementary Figure 1 ROC curve analysis for arthralgia miRNA expression levels. ROC curves showing the prediction accuracy for Arthralgia using combinations of miRNA expression levels. AUC calculated with 95% confidence intervals.

Supplementary Figure 2 Expression of miR-146, miR-125a/b, miR-16, miR-223, and miR-155 between RA patients, Arthralgia, and HC individuals. miRNA expression was determined by Multiplex Circulating miRNA Assay. Graphs show mean fluorescence intensity (MFI) for RA patients (N = 49) arthralgia (N = 10), and HC individuals (N = 20) represented as mean ± SEM. **p ≤ 0.01, *p < 0.05 as determined by Mann-Whitney *U* test.
