# Supplementary material for: Serum miRNA Signature in Rheumatoid Arthritis and “At-Risk Individuals”
Source: Front Immunol. 2021 Mar 3;12:633201. doi: 10.3389/fimmu.2021.633201 (PMC7966707; doi:10.3389/fimmu.2021.633201)

Supplementary Figure 1

Combined ROC Curves (2 miRNA) – Arth v HC

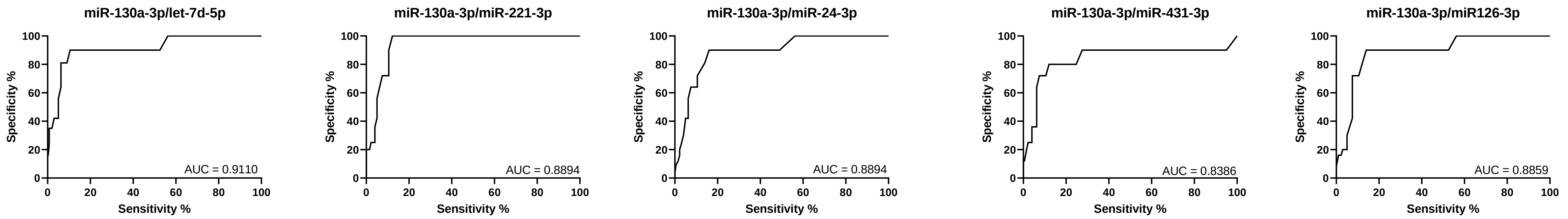

Combined ROC Curves (3 miRNA) – Arth v HC

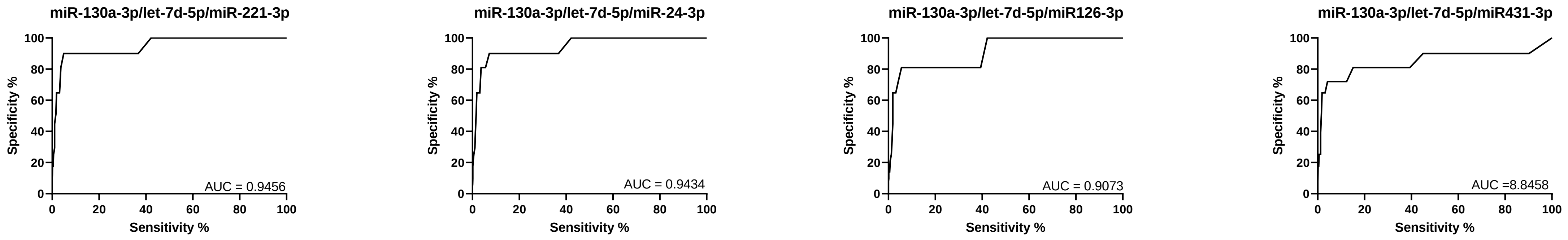

Supplementary Figure 2

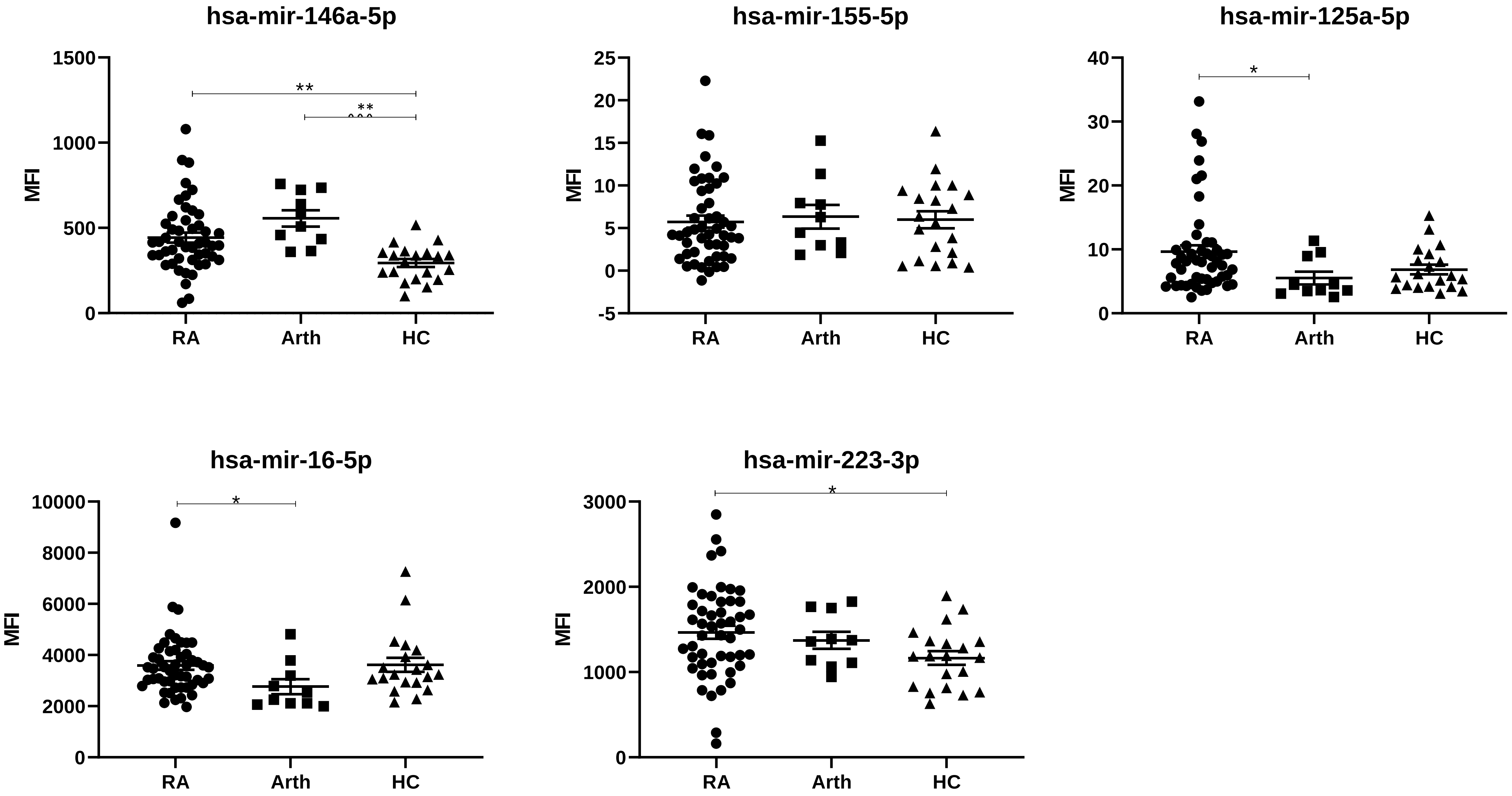

Supplement: Supplementary Figure 1 — ROC curve analysis for arthralgia miRNA expression levels. ROC curves showing the prediction accuracy for Arthralgia using combinations of miRNA expression levels. AUC calculated with 95% confidence intervals. [file DataSheet_1.pdf]
