# Supplementary material for: Serum miRNA Signature in Rheumatoid Arthritis and “At-Risk Individuals”
Source: Front Immunol. 2021 Mar 3;12:633201. doi: 10.3389/fimmu.2021.633201 (PMC7966707; doi:10.3389/fimmu.2021.633201)
Supplement: Supplementary file 2 [file DataSheet_2.pdf]

Supplementary Table 1: Patient Baseline Clinical Parameters

Demographics	RA	Arthralgia	HC
N=Number	49	10	20
Age; mean, (range)	58 (27-83)	49 (27-68)	38(27-49)
Female/Male	36/13	8/2	13/7
DAS28, mean ± SD	4.42 ± 1.35	2.36 ± 1.03	N/A
VAS, mean ± SD	62.93 ± 27.67	55 ± 29.5	N/A
CRP, mean ± SD	23.55 ± 43.27	4.8 ± 3.8	N/A
ACPA pos/	25/24	10/10	N/A

DAS28, Disease activity score; CRP, C-reactive protein; ESR, erythrocyte sedimentation rate;.VAS; Visual Anlogue scale of patient pain. ACPA-anti-citrulliinated protein antibody.

Supplementary Table 2: Patient Clinical Parameters Pre- and Post-MTX

Demographics	Pre-MTX N=18	Post-MTX N=18
N=number	18	18
Age	54 (27-75)	54(27-75)
Female/Male	15/3	15/3
DAS28, mean ± SD	4.43 ± 1.37	3.78 ± 1.34
VAS, mean ± SD	58.46 ± 32.19	53.11 ± 29.12
CRP, mean ± SD	26.97 ± 56.07	7.5 ± 10.84
ACPA pos/neg	10/8	10/8
Good response (number)	N/A	6
Moderate response (number)	N/A	5
No response (number)	N/A	7

DAS28, Disease activity score; CRP, C-reactive protein; ESR, erythrocyte sedimentation rate; VAS, Visual Analogue scale of patient pain. ACPA-anti-citrulliinated protein antibody

Supplementary Table 3. **The Multiplex Circulating miRNA Immunology Fixed Panel**

miRbase 20 name	miRNA sequence
hsa-let-7b-5p	ugagguaguagguugugugguu
hsa-let-7d-5p	agagguaguagguugcauaguu
hsa-let-7e-5p	ugagguaggagguuguauaguu
hsa-let-7g-5p	ugagguaguaguuuguacaguu
hsa-let-7i-5p	ugagguaguaguuugugcuguu
hsa-miR-10a-5p	uacccuguagauccgaauuugug
hsa-miR-122-5p	uggagugugacaaugguguuug
hsa-miR-1246	aauggauuuuuggagcagg
hsa-miR-125a-5p	ucccugagacccuuuaaccuguga
hsa-miR-126-3p	ucguaccgugaguaauaaugcg
hsa-miR-129-5p	cuuuuugcggucugggcuugc
hsa-miR-130a-3p	cagugcaauguuaaaagggcau
hsa-miR-132-3p	uaacagucuacagccauggucg
hsa-miR-140-3p	uaccacaggguagaaccacgg
hsa-miR-142-3p	cauaaaguagaaagcacuacu
hsa-miR-145-5p	guccaguuuucccaggaaucccu
hsa-miR-146a-5p	ugagaacugaauuccauggguu
hsa-miR-150-5p	ucucccaacccuuguaccagug
hsa-miR-151a-5p	ucgaggagcucacagucuagu
hsa-miR-154-3p	aaucauacacgguugaccuauu
hsa-miR-155-5p	uuaaugcuaaucgugauaggggu
hsa-miR-15a-5p	uagcagcacauaaugguuugug
hsa-miR-15b-5p	uagcagcacaucaugguuuaca
hsa-miR-16-5p	uagcagcacguaaauauuggcg
hsa-miR-16-2-3p	ccaauauuacugugcugcuuua
hsa-miR-17-5p	caaagugcuuacagugcagguag
hsa-miR-181a-5p	aacauucaacgcugucggugagu
hsa-miR-181b-5p	aacauucauugcugucgguggguu
hsa-miR-185-5p	uggagagaaaggcaguuccuga
hsa-miR-192-5p	cugaccuaugaauugacagcc
hsa-miR-196a-5p	uagguaguuucauguuguuggg
hsa-miR-200a-3p	uaacacugucugguaacgaugu
hsa-miR-203a	gugaaauguuuaggaccacuag
hsa-miR-205-5p	uccuucauuccaccggagucug
hsa-miR-20a-5p	uaaagugcuuauagugcagguag
hsa-miR-20b-5p	caaagugcucauagugcagguag
hsa-miR-21-5p	uagcuuaucagacugauguuga
hsa-miR-210-3p	cugugcgugugacagcggcuga
hsa-miR-22-3p	aagcugccaguugaagaacugu
hsa-miR-221-3p	agcuacauugucugcuggguuuc
hsa-miR-223-3p	ugucaguuugucaaauacccca
hsa-miR-23a-3p	aucacauugccagggauuucc
hsa-miR-24-3p	uggcucaguucagcaggaacag
hsa-miR-26a-5p	uucaaguaauccaggauaggcu

hsa-miR-29a-3p	uagcaccaucugaaaucgguua
hsa-miR-29b-3p	uagcaccauuugaaaucaguguu
hsa-miR-29c-3p	uagcaccauuugaaaucgguua
hsa-miR-30a-5p	ugaccgauuucuccugguguuc
hsa-miR-30b-5p	uguaaacauccuacacucagcu
hsa-miR-320a	aaaagcuggguugagagggcga
hsa-miR-320d	aaaagcuggguugagagga
hsa-miR-339-5p	ucccuguccuccaggagcucacg
hsa-miR-33a-5p	gugcauuguaguugcauugca
hsa-miR-342-3p	ucucacacagaaaucgcacccgu
hsa-miR-34a-5p	uggcagugucuuagcugguugu
hsa-miR-375	uuuguucguucggcucgcguga
hsa-miR-376b-3p	aucauagaggaaaauccauguu
hsa-miR-422a	acuggacuuagggucagaaggc
hsa-miR-429	uaauacugucugguaaaaccgu
hsa-miR-431-3p	caggucgucuugcagggcuucu
hsa-miR-451a	aaaccguuaccauuacugaguu
hsa-miR-486-5p	uccuguacugagcugccccgag
hsa-miR-494-3p	ugaaacauacacgggaaaccuc
hsa-miR-523-5p	cucuagagggaagcgcuuucug
hsa-mir-744-5p	ugcggggcuagggcuaacagca
hsa-miR-885-5p	uccauuacacuacccugccucu
hsa-miR-92a-3p	uauugcacuugucccggccugu
hsa-mir-93-5p	caaagugcuguucgugcagguag
x-control	x-control
blank	none
